# The adjuvant BcfA activates antigen presenting cells through TLR4 and supports T_FH_ and T_H_1 while attenuating T_H_2 gene programming

**DOI:** 10.3389/fimmu.2024.1439418

**Published:** 2024-08-29

**Authors:** Mohamed M. Shamseldin, Kaitlin A. Read, Jesse M. Hall, Jasmine A. Tuazon, Jessica M. Brown, Myra Guo, Yash A. Gupta, Rajendar Deora, Kenneth J. Oestreich, Purnima Dubey

**Affiliations:** ^1^ Departments of Microbial Infection and Immunity, The Ohio State University, Columbus, OH, United States; ^2^ Departments of Microbiology, The Ohio State University, Columbus, OH, United States; ^3^ Department of Microbiology and Immunology, Faculty of Pharmacy, Helwan University-Ain Helwan, Helwan, Egypt; ^4^ Pelotonia Institute for Immuno-Oncology, The Ohio State University, Columbus, OH, United States; ^5^ Comprehensive Cancer Center, The Ohio State University, Columbus, OH, United States

**Keywords:** adjuvants, antigen presenting cells, BcfA, T cell polarization, pattern recognition receptors

## Abstract

**Introduction:**

Adjuvants added to subunit vaccines augment antigen-specific immune responses. One mechanism of adjuvant action is activation of pattern recognition receptors (PRRs) on innate immune cells. *Bordetella* colonization factor A (BcfA); an outer membrane protein with adjuvant function, activates T_H_1/T_H_17-polarized immune responses to protein antigens from *Bordetella pertussis* and SARS CoV-2. Unlike other adjuvants, BcfA does not elicit a T_H_2 response.

**Methods:**

To understand the mechanism of BcfA-driven T_H_1/T_H_17 vs. T_H_2 activation, we screened PRRs to identify pathways activated by BcfA. We then tested the role of this receptor in the BcfA-mediated activation of bone marrow-derived dendritic cells (BMDCs) using mice with germline deletion of TLR4 to quantify upregulation of costimulatory molecule expression and cytokine production in vitro and in vivo. Activity was also tested on human PBMCs.

**Results:**

PRR screening showed that BcfA activates antigen presenting cells through murine TLR4. BcfA-treated WT BMDCs upregulated expression of the costimulatory molecules CD40, CD80, and CD86 and produced IL-6, IL-12/23 p40, and TNF-α while TLR4 KO BMDCs were not activated. Furthermore, human PBMCs stimulated with BcfA produced IL-6. BcfA-stimulated murine BMDCs also exhibited increased uptake of the antigen DQ-OVA, supporting a role for BcfA in improving antigen presentation to T cells. BcfA further activated APCs in murine lungs. Using an *in vitro* T_H_ cell polarization system, we found that BcfA-stimulated BMDC supernatant supported T_FH_ and T_H_1 while suppressing T_H_2 gene programming.

**Conclusions:**

Overall, these data provide mechanistic understanding of how this novel adjuvant activates immune responses.

## Introduction

Many approved vaccines are comprised of purified antigens admixed with an adjuvant, referred to as subunit vaccines. The inclusion of immune stimulatory adjuvants in vaccine formulations bolsters immune responses versus antigens alone, supports dose sparing, reduced frequency of administration (1, 2), and improves the stability and pharmacokinetics of the antigens leading to an increased *in vivo* half-life (2). Another notable advantage is the ability of the adjuvant to shape the phenotype of the resulting cellular and humoral responses (1).

Alum (aluminum hydroxide or aluminum phosphate) was the first adjuvant to be licensed for human use more than a century ago. For seven decades, it was the only adjuvant used in FDA- and EMA-approved vaccines administered to protect against a multitude of infectious diseases including hepatitis, pertussis, and diphtheria (3, 4). In the late 1990s, other adjuvants were approved for addition to previously licensed human vaccines (3). The first of these was the oil-in-water emulsion MF59 added to a trivalent influenza vaccine, FLUAD (5, 6). The more recently described AS01-AS04 family of adjuvants are TLR4 ligands and aim to maximize the immune response while maintaining tolerability using a mix of classical adjuvants and other immunostimulatory molecules (3). These are included in vaccines against shingles (7), malaria (8), pandemic influenza (9), HBV and HPV (10). The oligonucleotide cytosine phosphoguanosine 1018 (CpG 1018), is a TLR9 agonist included in a hepatitis B vaccine (11–13).

These approved adjuvants have different mechanisms of action and are safe and effective at generating T cell and antibody-mediated responses. Notably, however, they all elicit mixed T_H_1/T_H_2-polarized immunity (14). In contrast, natural immune responses to infection with most viral and bacterial pathogens elicit T_H_1/T_H_17-polarized T cell and antibody responses, which are correlated with sustained protection against disease and infections in both humans and mouse models (15–19). Recent studies (20) also highlight the importance of tissue-resident memory T (T_RM_) and B cell responses which provide sustained protection at barrier sites (21–24). Therefore, there is a need for safe novel adjuvants that elicit T_H_1/T_H_17_-_polarized immune responses, and also generate tissue-resident memory. To rationally design such adjuvants, it is critical to understand their mechanism of action. We previously identified *Bordetella* colonization factor A (BcfA), a bacterial protein of the Gram-negative pathogen *Bordetella bronchiseptica*, and we have reported that it functions as an adjuvant *in vivo* (25). Systemic administration of BcfA-containing vaccines elicited T_H_1- and T_H_17-polarized CD4^+^ and T_H_1-polarized CD8^+^ T cell responses (25), T follicular helper (T_FH_) cells, and T_H_1-skewed antibody responses (20) alone and in combination with alum. Mucosal immunization with antigens mixed with BcfA elicited CD4^+^IL-17^+^ T_RM_ cells in the lungs and nose (20, 26). Importantly, when BcfA was mixed with alum-containing approved and experimental vaccines, T_H_2 responses were attenuated while T_H_1 and T_H_17 responses were sustained or amplified (20, 25), suggesting that BcfA can override the T_H_2 polarized responses elicited by alum. This unique property therefore supports the potentially broad applicability of BcfA as an adjuvant for bacterial and viral pathogens where T_H_1 and T_H_17 responses are important for protection against infection and disease.

Here, we investigated the mechanism of BcfA activation of immune responses. We report that BcfA activates bone marrow-derived dendritic cells (BMDCs) through the pattern-recognition receptor TLR4. We observed dose-dependent upregulation of costimulatory molecules CD40, CD80 and CD86 on wildtype (WT) BMDCs and the production of innate cytokines IL-6, TNFα and IL-12/23 p40. BcfA-activated BMDCs more efficiently processed and presented the model antigen DQ-OVA. Furthermore, BcfA-stimulated BMDC conditioned medium supported differentiation of T_FH_-like and T_H_1 CD4^+^ T cells in an *in vitro* culture system, demonstrating the ability of BcfA to shape therapeutically beneficial T cell responses. These results provide insight regarding the TLR-dependent mechanism of action of this novel adjuvant and suggest that inclusion of BcfA in next-generation vaccine formulations may represent a promising avenue to enhance protective long-term immunity.

## Materials and methods

### BcfA formulation

BcfA was produced and purified as described previously (27). Residual LPS was removed using MustangQ filters (Cytiva Life Sciences, Inc, Marlborough, MA, catalog no. MSTGXT25Q16) or polymyxin B agarose (Sigma-Aldrich, St. Louis MO, catalog no. P1411). Endotoxin was quantified [(LAL Chromogenic Endotoxin Quantitation Kit, Thermo Fisher Scientific, catalog no. 88282) and was ≤150 pg/µg protein.

### PRR screening

HEK-Blue cells overexpressing murine TLR4 (catalog no. hkb-mtlr4) or murine TLR2 (catalog no. hkb-mtlr2) (InvivoGen, Inc. San Diego, CA) were stimulated with 1 or 5 µg/mL BcfA for 24hr in duplicate wells. Production of the co-expressed secreted embryonic alkaline phosphatase (SEAP) reporter was quantified. Minimal LPS (minLPS, 0.2ng/mL) in the BcfA preparation was used as negative control and purified LPS (100 µg/mL) (eBioscience, San Diego, CA catalog. no 00-4976-93) was used as positive control for stimulation.

### Mice

All experiments were reviewed and approved by The Ohio State University (OSU) Institutional Animal Care and Use Committee (Protocol number 2017A00000090). C57BL/6J, TLR4 knockout mice on the C57BL/6 background were obtained from Jackson Laboratories and bred in our facility. Tail DNA from mice was genotyped by Transnetyx, Inc. (Cordova, TN) using validated probe sets to confirm genotypes.

### Differentiation of murine bone marrow derived dendritic cells

Murine BMDCs were prepared according to published protocols (28). Briefly, bone marrow was isolated, dissociated into a single cell suspension, and red blood cells were lysed with ACK lysing buffer (Gibco Ref A10492). The cell suspension was resuspended in RPMI1640 (Gibco) + 10% fetal bovine serum (FBS, Sigma-Aldrich F4220), 10µg/mL gentamicin, 5x10^-5^M ß-mercaptoethanol and 40 ng/mL GM-CSF (R&D Systems, Minneapolis, MN, catalog no. 415-ML-020)) and seeded in 10 cm^2^ non-tissue culture treated petri dishes (VWR catalog no. 25384-342) at a density of 5-10 x10^6^ cells/plate. Half of the medium was replaced every 2 days with the addition of fresh GM-CSF. On day 6-7 post-differentiation, BMDCs were transferred to 6-well tissue culture-treated plates (Falcon catalog no. 353046) at a density of 0.5-1 x10^6^ cells/well. The next day, the cells were stimulated with various concentrations of BcfA for 20-24hr. Supernatant was collected for ELISA analysis and the cells were harvested for flow cytometry.

### Collection of human PBMCs and stimulation with BcfA

Peripheral blood was collected from adult human donors under a protocol approved by The Ohio State University Institutional Review Board (Protocol numbers 2020H0404 and 2021H0179). Whole blood was collected in EDTA treated tubes. PBMCs were separated from whole blood on a Percoll gradient (Cytiva, catalog no. 17144003), and cryo-preserved at -80°C. Cells were thawed and cultured in RPMI + 10% human AB serum (Sigma Aldrich, catalog no. H4522). To determine TLR4 expression, PBMCs were stained with α-human TLR4 (Thermo Fisher Scientific catalog no. 12-9041-80) antibody. Cells were analyzed on a Cytek Aurora spectral flow cytometer. Fluorescence minus one (FMO) controls were used for gating. To test cytokine production, 1-2x10^6^ cells from individual donors were stimulated for 20-24hr and the supernatant was tested by ELISA.

### Enzyme-linked immunosorbent assay for innate cytokines

The production of murine TNF-α, IL-6, IL-12/23 p40 common γ chain and IL-12 was quantified by a sandwich ELISA according to the manufacturer’s instructions (Life Technologies or BioLegend). Human cytokines IL-6 and TNF-α were quantified using Quantikine ELISA kits (R&D Systems). Plates were read at A_450_ on a SpectraMax i3x^®^ plate reader and concentrations were calculated based on the standard curve.

### DQ-OVA uptake and processing

BMDCs were plated in 6 well plates at 1 x10^6^ cells/well and stimulated with BcfA or cultured with medium alone. At 24 hr post-stimulation, DQ-OVA (InvivoGen, Inc. catalog no. D12053) was added at a concentration of 10 µg/mL for 45 min. The cells were then washed and harvested to quantify expression of CD11c, MHC-Class II and DQ-OVA by flow cytometry.

### Immunization of mice

Mice were lightly anesthetized with 2.5% isoflurane/O_2_ for immunization. To stimulate APCs in the lungs, mice were immunized intranasally with 10 µg BcfA or LPS-EB VacciGrade™ (InvivoGen, catalog no. vac-3pelps) (a TLR4 agonist used as a positive control) in 50 µL divided between both nares. Lungs were harvested 24 hr post-inoculation.

### Tissue dissociation and flow cytometry

BMDCs were harvested at 20-24hr post-stimulation and were washed with cold PBS prior to staining with Live/Dead Zombie NIR fixable viability dye (BioLegend, catalog no. 423105) for 30 min at 4°C. Cells were then washed twice with PBS supplemented with 1% heat-inactivated FBS (1% FBS) (FACS buffer) and resuspended in Fc Block (α-CD16/CD32 antibody, clone 93) (eBioscience, catalog no. 14-0161-86) at 4°C for 5 min before staining with a mixture of the following Abs for 20 min at 4°C: CD11c e450 (clone N418; Invitrogen, catalog no.48-0114-82), MHC Class II I-A/I-E BV785 (clone M5/114.15.2; Biolegend, catalog no.107645), CD40-APC (clone 1C10), CD80 PerCP-Cy5.5 (clone 16-10A1; Biolegend, catalog no.104722) and CD86 FITC (clone GL1; eBioscience, catalog no.11-0862-85).

Lungs were processed, digested (mouse lung dissociation kit, Miltenyi Biotec, catalog no. 130-095-927), and mechanically disrupted (gentleMACS) into a single-cell suspension followed by RBC lysis.Cells were washed with cold PBS and stained with Live/Dead Zombie NIR fixable viability dye (BioLegend, catalog no. 423105) for 30 min at 4°C, then washed with PBS/1% FBS and resuspended in Fc Block at 4°C for 5 min. Lung cells were stained with a mixture of the following antibodies for 20 min at 4°C: CD11b APC (clone M1/70; Invitrogen, catalog no.17-0112-82), CD11c e450 (clone N418; Invitrogen, catalog no.48-0114-82), MHC Class II I-A/I-E BV785 (clone M5/114.15.2; Biolegend, catalog no.107645), CD45 PE (clone 30-F11; BD Biosciences, catalog no.553081), CD40 PE-CF594 (clone 3/23; BD Biosciences, catalog no.562847), CD80 PerCP-Cy5.5 (clone 16-10A1; Biolegend, catalog no.104722) and CD86 FITC (clone GL1; eBioscience, catalog no.11-0862-85).

For intracellular cytokine staining, cells were first incubated in complete IMDM (cIMDM; IMDM [Life Technologies], 10% FBS [26140079, Life Technologies], 1% Penicillin-Streptomycin [Life Technologies], and 0.05% (50 mM) 2-ME [Sigma-Aldrich]) with eBioscience protein transport inhibitors (PTI; catalog no. 00-4980-93, Invitrogen) for 4 hr. For cell surface marker staining, samples were pre-incubated for 5 min at 4°C with TruStain FcX™ (α-mouse CD16/32) Fc block (clone 93; catalog no. 101320, BioLegend). Samples were then stained for extracellular markers in the presence of Fc block for 30 min at 4°C protected from light using the following antibodies: α-CD4 (PerCP-Cy5.5; 1:100; clone GK1.5; catalog no. 100434, BioLegend) and Ghost Dye (V510; 1:400; catalog no. 50-105-2992, Tonbo Biosciences). Cells were then washed twice with FACS buffer and were fixed and permeabilized using the eBioscience Foxp3 transcription factor staining kit (Thermo Fisher Scientific, catalog no. 00-8333-56) overnight at 4°C.

Following fixation, cells were washed once with the 1X eBioscience permeabilization buffer (Thermo Fisher Scientific). Recombinant Mouse IL-21R Fc Chimera primary antibody (catalog no. 596-MR, R&D) was diluted 1:5 in 1X eBioscience permeabilization buffer and 100 µL was added per well to incubate for 30 min at 4°C protected from light. Cells were washed once with 1X eBioscience permeabilization buffer. Goat F(ab’)2 Anti-Human IgG - Fc secondary antibody (PE, catalog no. ab98596, Abcam) was diluted 1:31.25 in 1X eBioscience permeabilization buffer and 100 µL was added per well to incubate for 30 min at 4°C protected from light. For staining the remaining intracellular markers, cells were washed once with 1X eBioscience permeabilization buffer and then stained with the following antibodies in 1X eBioscience permeabilization buffer for 1hr at room temperature protected from light: α-Bcl6 (AF488; 1:20; clone K112-91; catalog no. BDB561524, BD Biosciences); α-Gata3 (PE-Cy7; 1:20; clone TWAJ; catalog no. 25-9966-42, Invitrogen); α-IL-4 (APC; 1:50; clone 11B11; catalog no. 504106, BioLegend); α-IFN-γ (APC-Cy7; 1:300; clone XMG1.2; catalog no. 505850, BioLegend), and α-T-bet (PacBlue; 1:50; clone 4B10; catalog no. 644808, BioLegend). Cells were washed twice with 1X permeabilization buffer and resuspended in FACS buffer for analysis.

Fluorescence minus one or isotype control antibodies were used as negative controls. After two washes, cells were resuspended in PBS/1% FBS and samples were collected on or BD FACS Symphony or Cytek Aurora spectral flow cytometer (Cytek Biosciences).

Analysis was performed using FlowJo software version 10.8.0. The number of cells within each population was calculated by multiplying the frequency of live singlets in the population of interest by the total number of cells in each sample.

### Preparing and assaying CD4^+^ T cells for lineage-defining transcription factors and effector cytokines

Naïve CD4^+^ T cells were purified from the spleen and lymph nodes of 5-8-week-old WT C57BL/6J mice using the BioLegend Mojosort kit. Cells were plated in 24-well plates on plate-bound anti-CD3 (5 μg/mL) and anti-CD28 (2μg/mL) in the presence or absence of IL-4 neutralizing antibody (11B11, BioLegend, 5μg/mL). After 16-20hr, medium from non-stimulated control (NS Ctrl) or BcfA-treated BMDCs was added to the well, with or without IL-4-neutralizing antibody. Where indicated, IL-6-neutralizing antibody (MP5-20F3, BioLegend, 10µg/mL) was also added. Cells were cultured for an additional 48hr before harvest and subsequent analysis.

RNA was isolated using the Macherey-Nagel Nucleospin kit per the manufacturer’s instructions, and complementary DNA (cDNA) was synthesized using the SYBR Superscript IV First Strand Synthesis System with oligo dT primers (Thermo Fisher). qRT-PCR reactions were run on the CFX Connect (BioRad) with 5-20ng of cDNA, using the SYBR Select Mastermix for CFX (ThermoFisher) and the following primers:

*Bcl6* forward: 5’-CCAACCTGAAGACCCACACTC-3’, *Bcl6* reverse: 5’-GCGGACATGGCTCTTCAGAGTC-3’; *Il21* forward: 5’-TGGATCCTGAACTTCTATCAGCTCC -3’, *Il21* reverse: 5’- AGGCAGCCTCCTCCTGAGC -3’; *Tbx21* forward: 5’- GTGACTGCCTACCAGAACGC -3’, *Tbx21* reverse: 5’- AGGGGACACTCGTATCAACAG -3’; *Ifng* forward: 5’- CTACCTTCTTCAGCAACAGC -3’, *Ifng* reverse: 5’- GCTCATTGAATGCTTGGCGC -3’. Data were normalized to *Rps18* and are presented relative to either *Rps18* or the control sample, as noted.

### Statistical analysis

Data were analyzed using GraphPad Prism by the methods described in each figure legend.

## Results

### Activation of murine BMDCs by BcfA is dependent on TLR4

We hypothesized that BcfA activates antigen-presenting cells (APCs) through a PRR and conducted an empirical PRR screening using the HEK-Blue system, where human embryonic kidney (HEK) cells were transfected with the SEAP (secreted embryonic alkaline phosphatase) reporter gene under the control of a promoter inducible by NF-κB and activator protein-1 (AP-1) and expressing murine TLR (mTLR) or NOD receptors. SEAP reporter production was quantified using a colorimetric assay. The screen showed activation of mTLR4 and mTLR2 (**Supplementary Figure S1A**). We then stimulated cells expressing TLR4 (HEK-mTLR4) and TLR2 (HEK-mTLR2) with 1 or 5 µg/mL BcfA. Maximal SEAP release was detected from HEK cells expressing murine TLR4 (HEK-mTLR4) following stimulation with 1 µg/mL BcfA (**Supplementary Figure S1B**) at levels comparable to purified LPS used as a positive control. SEAP production from HEK-mTRL2 cells was nearly maximal at 5 µg/mL BcfA stimulation (**Supplementary Figure S1C**) with lower SEAP production detected with 1 µg/mL BcfA stimululation. To mitigate any confounding effects of BcfA produced in *E. coli* as a bacterial recombinant protein, we utilized a stringent purification procedure to remove LPS (27). HEK-mTLR4 or HEK-mTLR2 cells incubated with this minimal LPS (≤150 pg/µg protein) (min LPS) did not produce SEAP.

**Figure 1:**
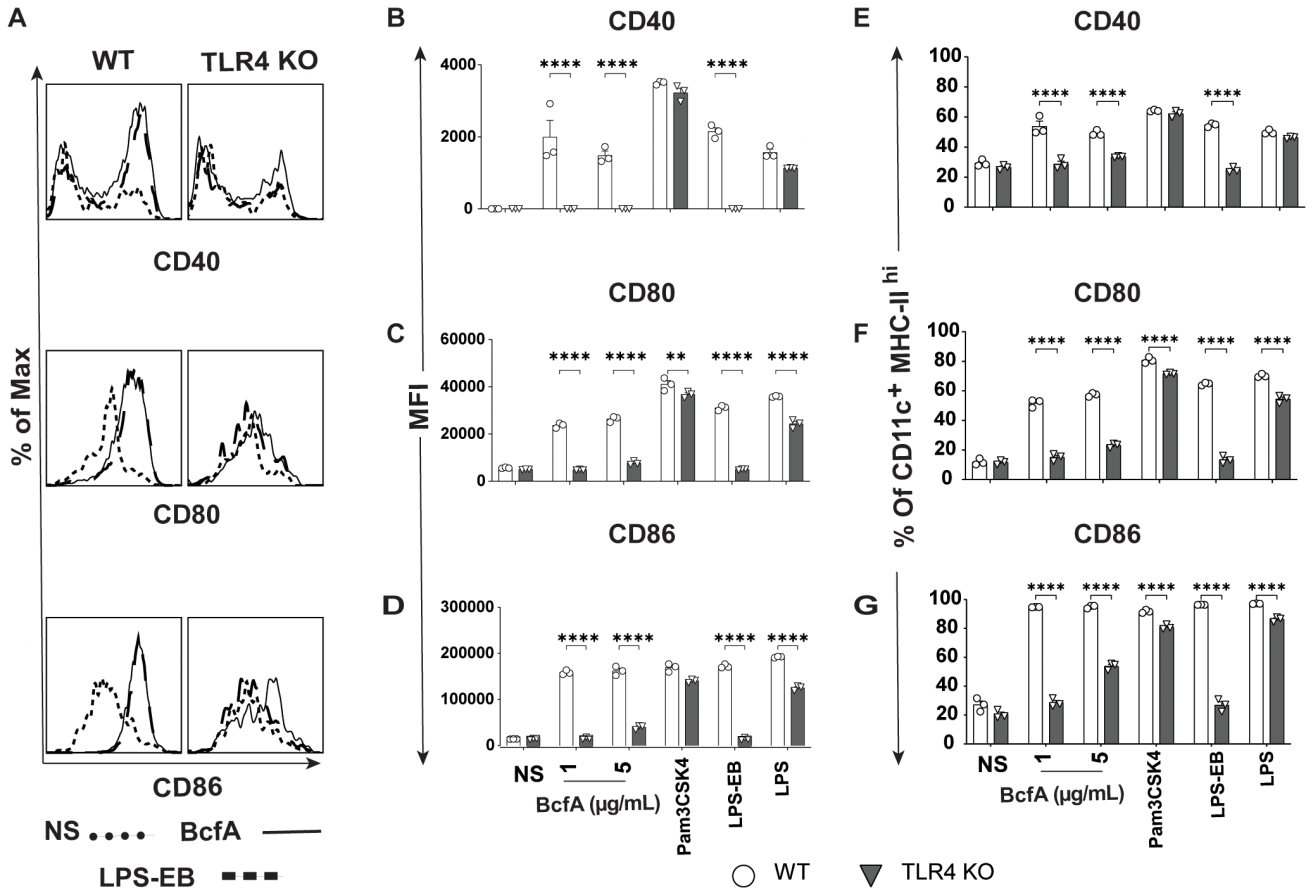
Costimulatory molecule expression is upregulated on BMDCs following BcfA stimulation. **(A)**. Representative overlays of CD40, CD80 and CD86 expression on WT and TLR4 KO BMDCs stimulated with 5 µg/mL BcfA. Median fluorescence intensity (MFI) expression of **(B)**. CD40, **(C)**. CD80 and **(D)**. CD86 and percentage of cells expressing **(E)**. CD40, **(F)**. CD80 and **(G)**. CD86 on WT and TLR4 KO BMDCs stimulated with 1 µg/mL and 5 µg/mL BcfA for 20-24 hr. LPS, Pam3CSK4 and LPS-EB were used as positive controls. Mean ± SEM of triplicate wells is shown. ^*^, p<0.05, ^**^, p<0.01, ^***^, p<0.001, ^****^, p<0.0001 by ANOVA. One experiment of 2.

We then evaluated the BcfA stimulatory activity in a more physiologically relevant system. We isolated bone marrow from WT C57BL/6 mice and TLR4 KO mice and differentiated BMDCs *in vitro*. The immature DCs were treated with BcfA (1 or 5 µg/mL) for 24 hr and analyzed for the expression of costimulatory molecules by flow cytometry. BMDCs were identified as a CD11c^+^ MHC-II^hi^ population (**Supplementary Figure S2**) (29) and were evaluated for the expression of costimulatory molecules by flow cytometry. Representative flow plots are shown in **Figure 1A**. The mean fluorescence intensity (MFI) expression of CD40 (**Figure 1B**), CD80 (**Figure 1C**), and CD86 (**Figure 1D**) and percentage of cells expressing CD40 (**Figure 1E**) CD80 (**Figure 1F**) and CD86 (**Figure 1G**) was upregulated in response to BcfA stimulation compared to no stimulation (NS) control. LPS-EB (de-O-acelylated lipooligosaccharide) (5 µg/mL) that has been used as an adjuvant *in vivo* (30) and purified *E. coli* LPS (100 ng/mL) were included as positive controls for TLR4, and PAM3Csk was used as a positive control to confirm that TLR4 KO BMDCs respond to stimulation. Both the MFI and percentage of cells that upregulated costimulatory molecule expression was reduced to background in BcfA-stimulated TLR4 KO BMDCs, suggesting that BcfA primarily functions through this PRR. We also tested activation of TLR2 KO BMDCs which showed that costimulatory molecule expression was not reduced by the absence of TLR2 (**Supplementary Figure S3**), suggesting that BcfA primarily functions through TLR4. Thus, we focused on TLR4 mediated activity of BcfA in the following studies.

### BcfA induces the production of T_FH_/T_H_1/T_H_17-polarizing cytokines by BMDCs

**Figure 2:**
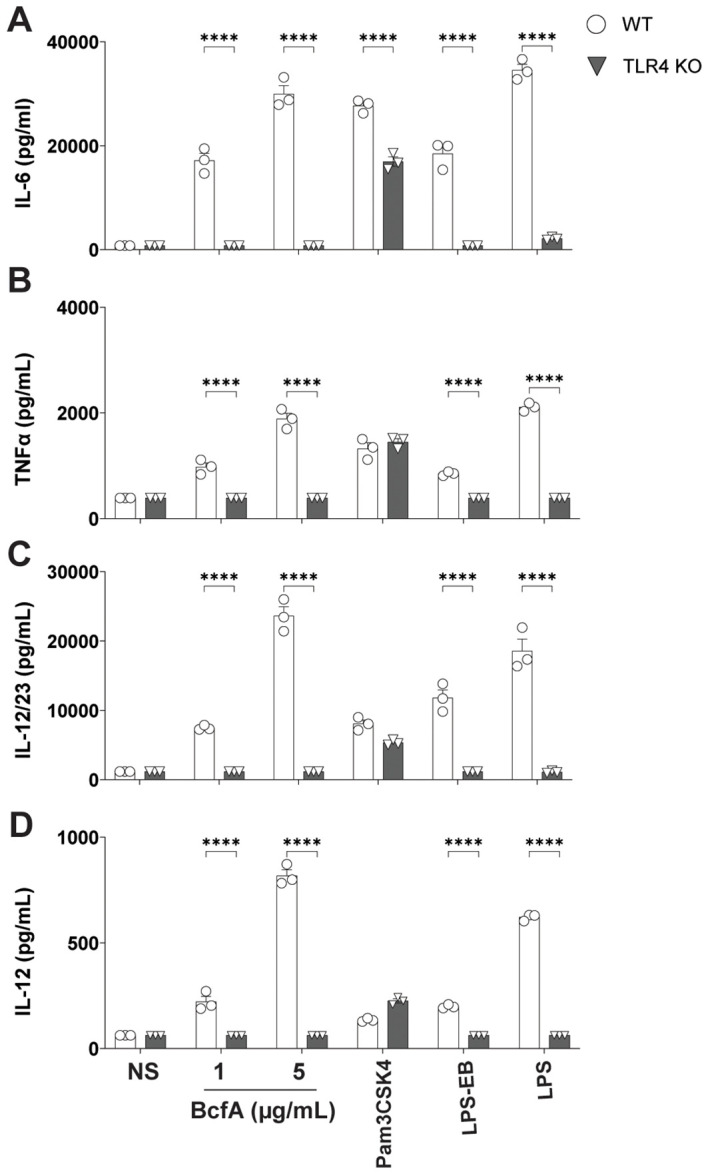
Production of T_FH_/T_H_1-polarizing innate cytokines following BcfA stimulation is dependent on TLR4. Expression of **(A)**. IL-6, **(B)**. TNF-α, **(C)**. IL-12/23 p40 and **(D)**. IL-12 by WT and TLR4 KO BMDCs stimulated with BcfA for 20-24 hr. LPS, Pam3CSK4 and LPS-EB were used as positive controls. Mean ± SEM of triplicate wells is shown. ^*^, p<0.05, ^***^, p<0.001, ^****^, p<0.0001 by ANOVA. One experiment of 2.

To determine the T cell responses that may be supported by APCs activated by BcfA, we quantified cytokine production from BcfA-stimulated BMDCs by ELISA. WT BMDCs produced IL-6 (**Figure 2A**), TNF-α (**Figure 2B**), IL-12/23 p40 (**Figure 2C**) and IL-12 (**Figure 2D**) at levels comparable to the positive control LPS. Production of all four cytokines was significantly reduced in TLR4 KO BMDCs. These results show that innate immune responses elicited by BcfA are mediated through TLR4 and may support T_H_1/T_H_17-polarized immune responses.

**Figure 3:**
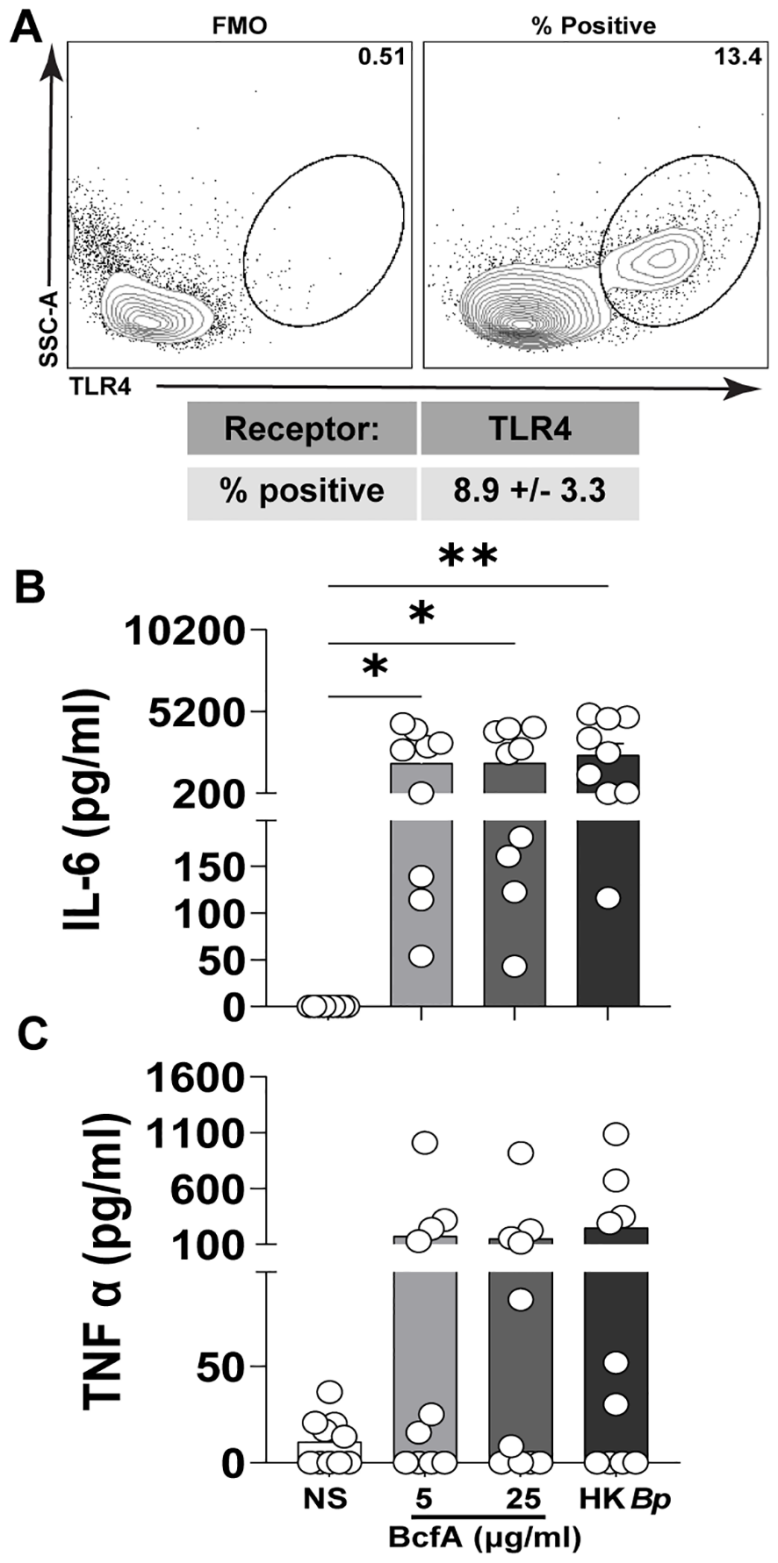
Human PBMCs produce IL-6 following stimulation with BcfA. **(A)**. The percentage of live PBMCs expressing TLR4 (N=5 adult donors) **(B)**. PBMCs from 10 adult donors were stimulated for 24 hr with the indicated concentrations of BcfA or heat-killed *B. pertussis* control (HK *Bp*). IL-6 and **(C)**. TNF-α in culture supernatant was evaluated by ELISA). ^*^, p<0.05, ^**^, p<0.01 by ANOVA.

We then tested whether BcfA activated human PBMCs. First, we confirmed expression of TLR4 on adult human PBMCs by flow cytometry (**Figure 3A**). We then stimulated PBMCs *in vitro* with BcfA and measured the levels of innate cytokines IL-6 and TNF-α present in the cell culture supernatant after 24 hr. We found that IL-6 was significantly induced in response to stimulation with 5 µg/mL or 25 µg/mL of BcfA (**Figure 3B**). Although TNF-α production was substantially increased upon stimulation with BcfA, differences were not statistically significant compared to unstimulated cells (**Figure 3C**). These data show that BcfA stimulation elicits T_H_1/T_H_17-polarizing innate cytokines by human cells.

### BcfA stimulation of BMDCs supports antigen uptake and processing

**Figure 4:**
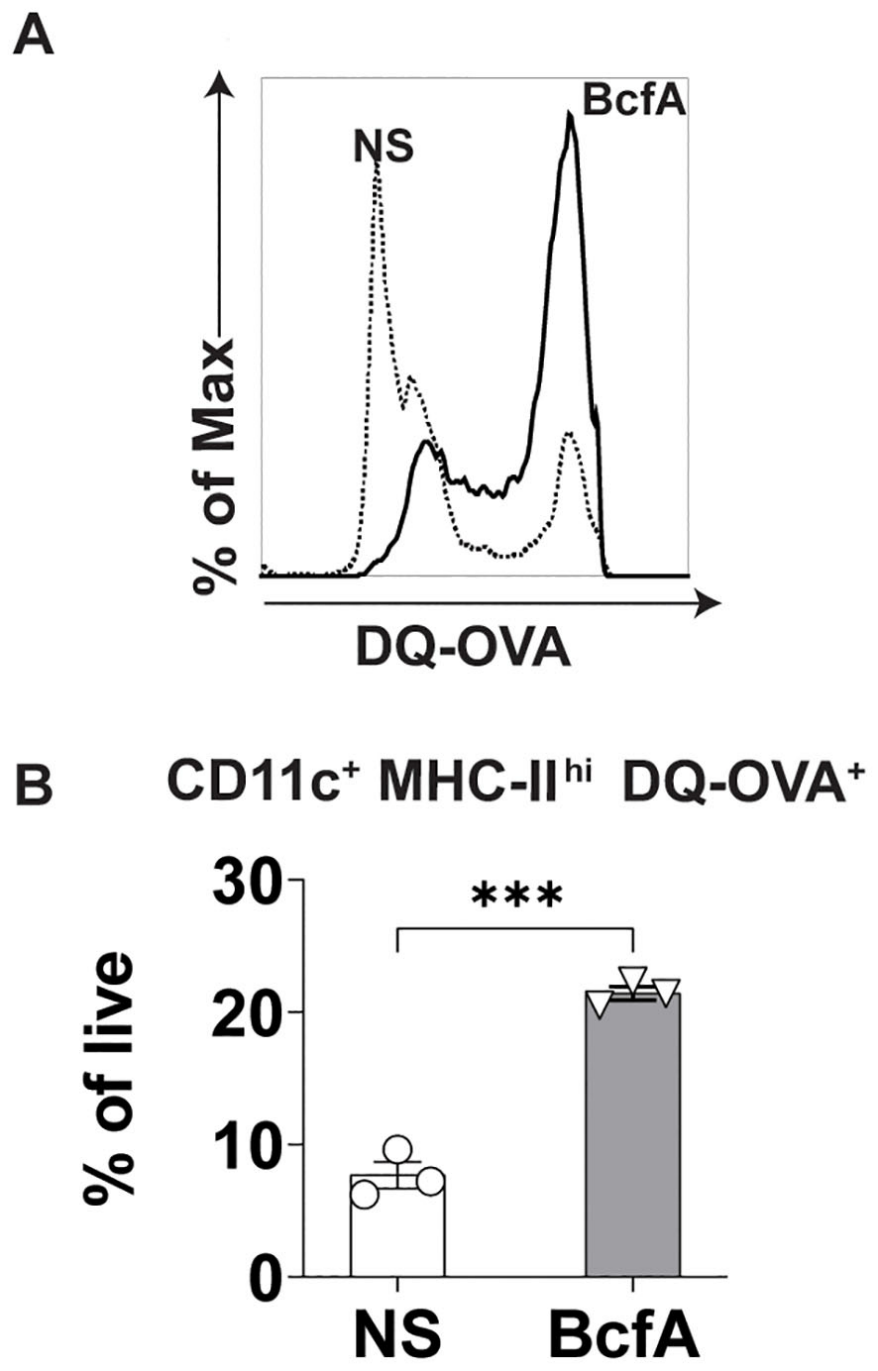
Increased uptake of DQ-OVA by BcfA-stimulated BMDCs. **(A)**. Representative overlays of non-stimulated (NS) and BMDCs stimulated with 5 µg/mL BcfA. **(B)**. CD11c^+^ MHC-II^+^ DQ-OVA^+^ BMDCs as % of live cells. Mean ± SEM of triplicate wells is shown. ^***^, p<0.001 by paired Student’s t-test. One experiment of 2.

Next, we evaluated whether BcfA stimulation of BMDCs would increase antigen uptake and processing of DQ-OVA, a chicken ovalbumin (OVA) conjugate that displays a bright green fluorescence only after proteolytic processing. We stimulated BMDCs with 5 µg/mL BcfA or media alone as a negative control for 24 hr at 37°C. The next day, cells were treated with DQ-OVA for 60 min. at 37°C. The level of green fluorescence was then quantified by flow cytometry as a proxy for antigen uptake and processing. BcfA-treated BMDCs took up more DQ-OVA than unstimulated BMDCs (**Figure 4A**). We also observed an increase in the percentage of BMDCs with green fluorescence (**Figure 4B**) compared to NS controls. Together, these data suggest that BcfA-activated BMDCs have an increased ability to uptake and process antigen.

### BcfA upregulates costimulatory molecule expression on CD11b^+^CD11c^+^ cells via TLR4 *in vivo*

**Figure 5:**
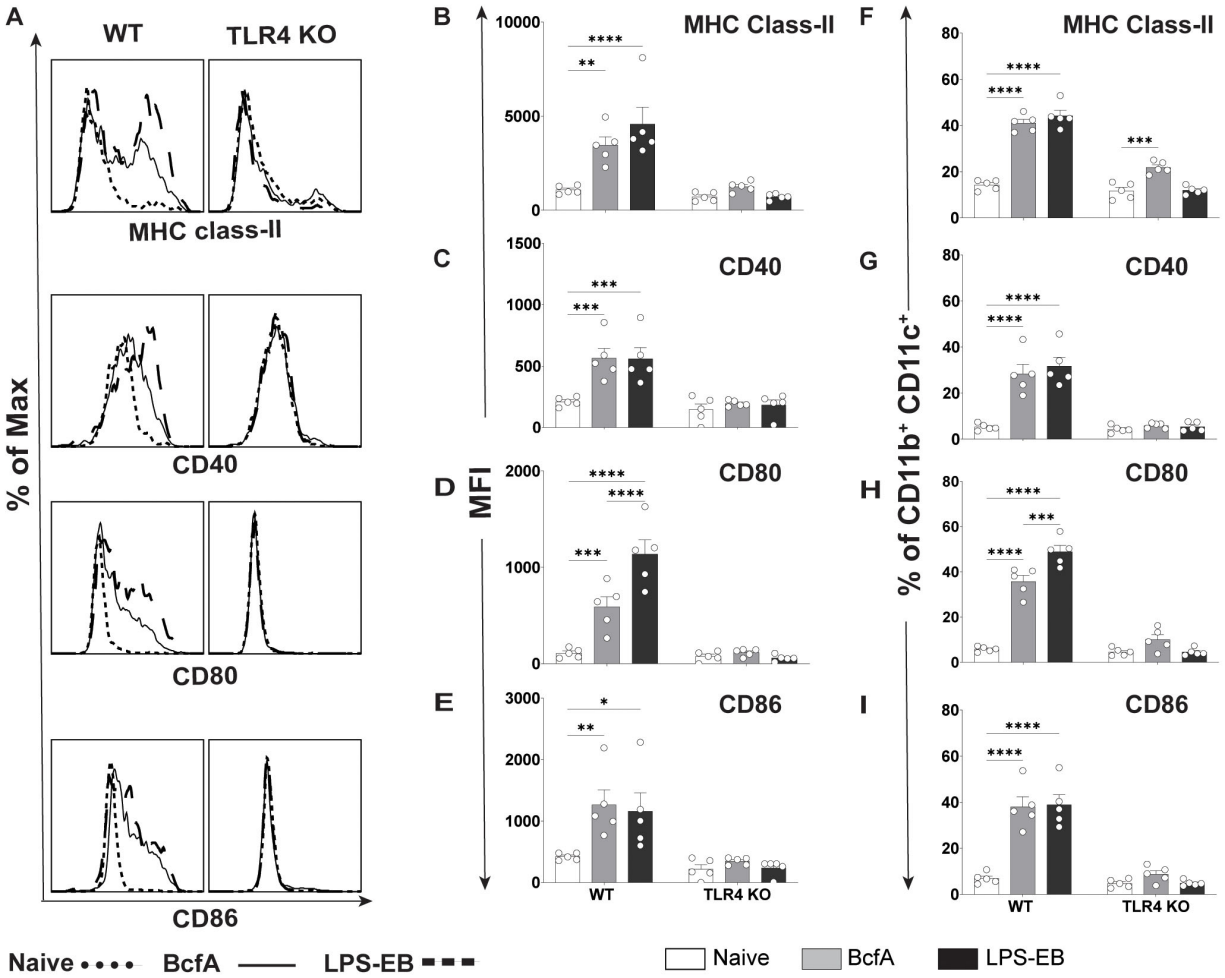
Upregulation of costimulatory molecule expression on lung CD11b^+^CD11c^+^ cells by BcfA is dependent on TLR4. **(A)**. Representative overlays of MHC class-II, CD40, CD80 and CD86 expression on lung cells from WT and TLR4 KO mice. Median fluorescence intensity (MFI) of **(B)**. MHC class-II, **(C)**. CD40, **(D)**. CD80 and **(E)**. CD86 and percentage of cells expressing **(F)**. MHC class-II, **(G)**. CD40, **(H)**. CD80, and **(I)**. CD86 on CD11b^+^ CD11c^+^ lung cells from WT and TLR4 KO mice, analyzed 24 hr post intranasal immunization with 10 µg of BcfA or LPS-EB. Mean ± SEM of 5 mice is shown. ^*^, p<0.05, ^**^, p<0.01, ^***^, p<0.001, ^****^, p<0.0001 by ANOVA. One experiment of 2.

To test whether BcfA stimulates lung cells, we administered 10 µg BcfA or LPS-EB intranasally to C57BL/6 and TLR4 KO mice. Lungs were harvested 24 hr later and evaluated for the expression of MHC Class II, CD40, CD80, and CD86 on the CD11b^+^ CD11c^+^ population (31) (**Figure 5A**). The gating strategy is shown in **Supplementary Figure S4**. MFI expression of MHC Class II (**Figure 5B**), CD40 (**Figure 5C**), CD80 (**Figure 5D**) and CD86 (**Figure 5E**) and the percentage of cells that upregulated expression of MHC Class II (**Figure 5F**), CD40 (**Figure 5G**), CD80 (**Figure 5H**) and CD86 (**Figure 5I**) increased on this cell population, demonstrating that BcfA efficiently activates putative antigen presented cells in the lungs. The MFI of costimulatory molecule expression was not upregulated on CD11b^+^ CD11c^+^ cells in TLR4 KO lungs (**Figures 5B–E**). The percentage of cells expressing MHC Class II compared to no stimulation increased in WT and TLR4 KO lungs (**Figure 5F**). In contrast the percentage of TLR4 KO lung cells expressing the costimulatory molecules CD40 (**Figure 5G**), CD80 (**Figure 5H**) and CD86 (**Figure 5I**) did not increase compared to no stimulation. These data further confirm TLR4 as the primary *bona fide* PRR for BcfA mediated activity.

### BcfA-stimulated BMDC-conditioned medium supports T_FH_ and T_H_1 cell programming

As part of pathogen-specific immune responses, naïve CD4^+^ T cells differentiate into effector subsets which perform specialized activities to orchestrate immune-mediated clearance of infection. Particularly critical for responses to intracellular pathogens like SARS-CoV-2 and primarily extracellular pathogens like *B. pertussis* are T helper 1 (T_H_1) and T follicular helper (T_FH_) populations. Of these, T_H_1 cells activate other immune populations by secreting pro-inflammatory cytokines such as IFN-γ, while T_FH_ cells produce IL-21 and provide help to B cells to promote antibody generation and humoral immunity (32, 33). We previously reported that intramuscular (i.m.) immunization with an acellular pertussis vaccine (aPV) containing BcfA elicited systemic T_H_1 responses *in vivo* (25). In our recent work (20), we established that immunization of mice with BcfA-adjuvanted SARS-CoV-2 spike protein supported production of IFN-γ-driven IgG2c spike protein-specific antibodies. Thus, we hypothesized that BcfA may support both T_H_1 and T_FH_ cell differentiation programs, and consequently productive anti-bacterial and anti-viral cell-mediated and humoral immune responses.

**Figure 6:**
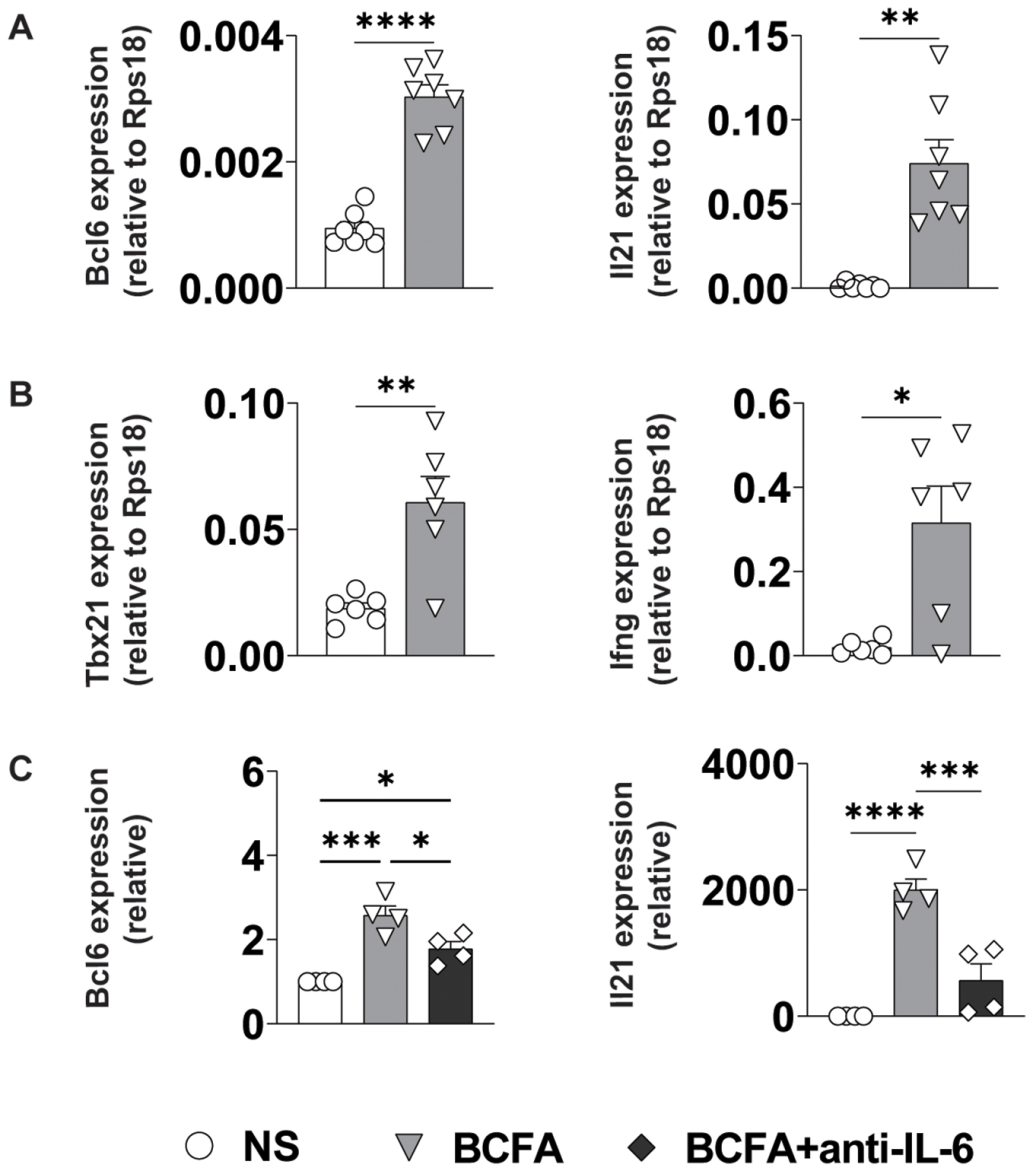
BcfA conditioned medium supports T_FH_ and T_H_1 cell polarization *in vitro*. qRT-PCR of T cells that were unstimulated (NS) or stimulated with BcfA conditioned medium with the addition of IL-4-neutralizing antibody. Data are presented relative to housekeeping gene *Rps18*. Data pooled from individual experiments with 4-7 samples per group. **(A)**. Bcl6 and IL21 expression, **(B)**. Tbx21 and IFN-γ expression, **(C)**. Bcl6 and IL21 expression alone or with anti-IL-6 neutralizing antibody. Mean ± SEM of 4 experiments is shown. ^*^, p<0.05, ^**^, p<0.01, ^***^, p<0.001, ^****^, p<0.0001 by paired Student’s t-test.

Effector CD4^+^ T cell differentiation is directed by a coordinated interplay between cell-intrinsic transcriptional networks and environmental cytokine signals that are often produced by antigen-presenting cells (34). To test whether the IL-6, IL-12, and IL-12/23 common chain cytokines in BcfA-stimulated supernatants would be sufficient to induce T_FH_ and/or T_H_1 differentiation, we stimulated naïve CD4^+^ T cells on plate-bound α-CD3/α-CD28 in the presence of cell-free supernatant from untreated BMDCs (NS) or those treated with 5 µg/mL BcfA for 72 hr. We then assessed the expression of key T_FH_ and T_H_1/genes via qRT-PCR. Consistent with our hypothesis, expression of genes for lineage-defining transcription factors and effector cytokines associated with T_FH_ (*Bcl6, Il21*; **Figure 6A**) and T_H_1 (*Tbx21* (T-bet), *Ifng*; **Figure 6B**) cells were elevated in cells cultured in the presence of BcfA-conditioned medium relative to NS controls.

As IL-6/STAT3 signaling is an established driver of T_FH_ differentiation, we determined whether the IL-6 in the culture medium was responsible for driving T_FH_ differentiation. We cultured cells in BcfA-treated or NS control medium in the presence or absence of IL-6-neutralizing antibody. Consistent with a role for IL-6 in promoting T_FH_ gene expression, we observed a significant reduction in the expression of both *Bcl6* and *Il21* when IL-6 was neutralized (**Figure 6C**).

**Figure 7:**
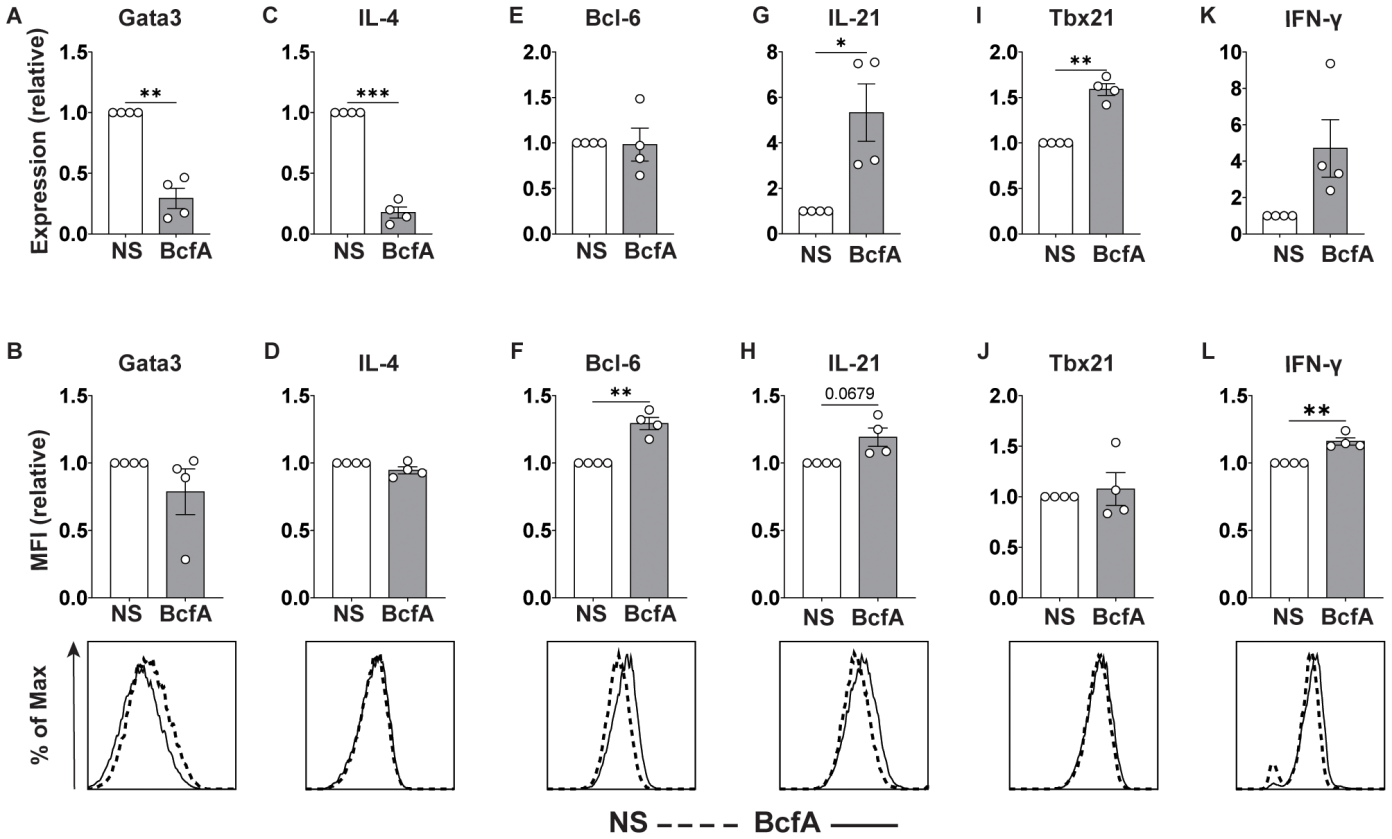
BcfA conditioned medium represses T_H_2 cell polarization *in vitro*. qRT-PCR and flow cytometry analysis of unstimulated (NS) T cells or stimulated with BcfA conditioned medium in the absence of IL-4-neutralizing antibody. qRT-PCR data are presented relative to housekeeping gene *Rps18*. Mean ± SEM of 4 experiments is shown. Expression of Gata3 **(A, B)**, IL-4 **(C, D)**, Bcl-6 **(E, F)**, IL-21 **(G, H)**, T-bet **(I, J)**, and IFN-γ **(K, L)**. ^*^, p<0.05, ^**^, p<0.01, ^***^, p<0.001 by paired Student’s t-test.

As standard T_FH_-like and T_H_1 culture conditions include the addition of anti-IL-4 neutralizing antibody which prevents inherent T_H_2 polarization (35), we also tested whether BcfA elicited T_FH_ or T_H_1 gene programming in the absence of IL-4-neutralization. Naïve T cells were differentiated *in vitro* without the addition of anti-IL-4 blocking antibody. We evaluated the expression of T cell subset-specific lineage-defining transcription factors and key cytokines by qRT-PCR and flow cytometry. Interestingly, BcfA reduced the RNA (**Figure 7A**) expression of the T_H_2 lineage-defining transcription factor Gata-3, while the protein level was unchanged (**Figure 7B**). Similarly, the RNA (**Figure 7C**) level of the T_H_2 effector cytokine IL-4 was reduced, while the protein (**Figure 7D**) expression was unchanged. In contrast, while transcript abundance for the T_FH_ lineage-defining transcription factor Bcl-6 was unchanged (**Figure 7E**), its protein expression was elevated (**Figure 7F**). Yet, transcript levels for the T_FH_ cytokine IL-21 were significantly elevated (**Figure 7G**), while protein expression was not significantly increased (**Figure 7H**). Finally, T_H_1 differentiation was elevated, as the RNA expression of the T_H_1-defining factor *Tbx21* (T-bet) was increased (**Figure 7I**), and the protein level was slightly but not significantly elevated (**Figure 7J**). RNA for the T_H_1 cytokine IFN-γ was unchanged (**Figure 7K**) while protein expression was increased (**Figure 7L**). Overall, these data suggest that BcfA both negatively regulates T_H_2 differentiation and positively regulates T_FH_ and T_H_1 programming.

## Discussion

The development and characterization of novel adjuvants is an active area of research, with the goal of defining adjuvants that elicit strong, sustained vaccine-mediated immune responses. Despite the decades-long use of alum as an adjuvant and the recent development of newer formulations, there remains a need for safe and effective adjuvants that elicit systemic and mucosal immunity.

Here, we investigated the mechanism of action of the adjuvant BcfA, which was discovered by our group and has demonstrated adjuvant activity in experimental vaccines against respiratory pathogens *B. pertussis* (25, 26) and SARS CoV-2 (20). The hallmark feature of BcfA that distinguishes it from FDA-approved and other experimental adjuvants is the absence of T_H_2 responses when BcfA is used as the single adjuvant (25) and strong attenuation of T_H_2 responses when BcfA is added to alum-adjuvanted vaccines (20, 26).

Furthermore, BcfA elicits T_H_1 and T_H_17 responses which are critical for protection against both bacterial and viral pathogens. Attenuation of T_H_2 immune responses may also reduce the risk of vaccine related adverse events such as antibody dependent enhancement of disease (ADE) and vaccine associated enhancement of respiratory disease (VAERD).

As BcfA is a protein, we hypothesized that it may activate immune responses through PRRs expressed on APCs. A PRR/NOD receptor screen identified both murine TLR4 and TLR2 as the receptors triggered on HEK-293 reporter cell lines following BcfA stimulation (**Supplementary Figure S1**). When the activity of BcfA was tested in murine BMDCs as a more physiological system, upregulation of costimulatory molecule expression (**Figure 1**) and production of innate cytokines (**Figure 2**) was significantly reduced in TLR4 KO BMDCs compared with WT BMDCs, but not in TLR2 KO BMDCs (**Supplementary Figure S3**). These data show that BcfA acts primarily through TLR4. BcfA-stimulated BMDCs showed uptake and processing of the model antigen DQ-OVA (**Figure 4**), suggesting that BcfA may also support T cell activation by amplifying antigen presentation. We pretreated BMDCs with BcfA prior to testing uptake of DQ-OVA. Coadministration of antigen and adjuvant may change the kinetics or magnitude of antigen uptake and processing and is a limitation of our experimental design.

To determine the potential utility of BcfA as an adjuvant for human vaccines, we tested cytokine production by human PBMCs stimulated with BcfA for 24 hr (**Figure 3**). Production of IL-6 was detected in supernatants of all samples tested, suggesting that human PBMCs stimulated with BcfA may support T_FH_ polarization of CD4^+^ T cells. Expression of the inflammatory cytokine TNF-α was variable between donors, and not significantly increased compared with unstimulated cells. It is important to note that we collected supernatant at 24 hr post-stimulation, which may be beyond the time of maximal TNF-α production in this context. Alternately, there may be autocrine consumption of TNF-α produced by the monocytes (36). Thus, the ability of BcfA to activate human cells suggests that BcfA will have adjuvant function when included in human vaccines.

Although bacterial LPS is the canonical ligand for TLR4, this PRR may also be activated by a wide array of bacterial proteins (37). Interestingly, triggering of the same receptor may elicit disparate immune phenotypes. The pneumococcal proteins DnaJ and Ply (38, 39) and the *M. tuberculosis* derived proteins RpfE and Rv0652 are TLR4 ligands that activate DCs and polarize the immune response towards the T_H_1/T_H_17 phenotype (40, 41), but elicit different innate cytokines. Collectively, these findings suggest that the type of immune response elicited by adjuvants depends both on the responding PRR as well as the properties of the ligand.

Adjuvants also activate the adaptive immune response by directly triggering PRRs on lymphocytes (42). Murine and human T cells express TLR4 (43). Thus, BcfA may directly activate T cells when delivered *in vivo* as part of a vaccine formulation, and thereby amplify the vaccine-elicited response. This potential function of BcfA is an area of future study for our group.

Using an *in vitro* T cell polarization system, we showed that the innate cytokines produced by BcfA-stimulated BMDCs support the differentiation of T_H_1 and T_FH_ cells while preventing T_H_2 differentiation in parallel (**Figure 6** and **Figure 7**). This result is in accordance with our previous report of the ability of BcfA to attenuate alum-induced T_H_2 responses when combined *in vivo* (20, 26). In addition, the IL-6-dependent polarization of T_FH_ cells we observed also provides a potential mechanistic explanation for the induction of IgG2c antibody production we observed in the serum and lungs of mice immunized with BcfA-containing vaccines.

Generation of mucosal immunity to respiratory pathogens is critical for providing sustained protection against infection (44, 45) and preventing subsequent transmission (46). For *B. pertussis*, the CD4^+^IL-17^+^ T cells generated during natural infection are essential for reducing bacterial colonization of the nose (45, 46). Immunization of baboons with the whole cell pertussis vaccine (wPV) also clears the infection from the nose (47). In contrast, acellular pertussis (aPV) vaccine immunization does not reduce nasal bacterial burden (48). Our recent paper (26) showed that mucosal delivery of BcfA-adjuvanted acellular pertussis vaccines elicited CD4^+^IL-17^+^ tissue-resident memory T cells (T_RM_) in the nose and reduced nasal bacterial burden. Although we detected production of the IL-12/23 p40 common γ chain by BcfA-stimulated BMDCs, suggesting that IL-17 may also be produced, we did not detect T_H_17 polarization *in vitro* (data not shown), suggesting that either the amount of IL-12/23 produced in BcfA supernatant is insufficient, or that additional factors such as such as TGF-β (49) that support T_H_17 generation are not replicated in the culture system.

In summary, this work provides a mechanistic understanding of the function of BcfA as an adjuvant and supports the utility of BcfA for use in human vaccines. Continued identification and validation of novel adjuvants that are safe, effective, and generate mucosal immunity is key to improving vaccine-mediated protection against infectious diseases.

## Data Availability

The original contributions presented in the study are included in the article/**Supplementary Material**. Further inquiries can be directed to the corresponding author.

## Supplementary material

Supplementary Figure 1 BcfA activates BMDCs and TLR2 and TLR4 expressing reporter cell lines. **(A)**. HEK-Blue™ cells expressing murine TLRs, and NOD1/2 were stimulated with BcfA (1µg/mL). SEAP reporter expression was detected using HEK-Blue™ Detection media. The average of duplicate wells is shown. **(B)**. HEK-Blue-mTLR4 and **(C)**. HEK-Blue-mTLR2 cells were stimulated with 1 and 5µg/mL of BcfA. NT = no treatment; min LPS = minimal LPS present in the BcfA preps. PAM3CSK4 and LPS were used as positive controls for TLR2 and TLR4 respectively. Mean ± SEM of triplicate wells is shown. ^****^, p<0.0001 by ANOVA. One experiment of 2.

Supplementary Figure 2 Gating strategy to identify activated BMDCs. Differentiated BMDCs were stained with antibodies specific for CD11c, MHC Class II, CD40, CD80 and CD86. Live, single cells were gated as CD11c^+^MHC-II^high^ cells. This double positive population from WT and TLR4 KO cells was evaluated for expression of CD40, CD80 and CD86 as shown.

Supplementary Figure 3 Upregulation of costimulatory molecule expression is unchanged in TLR2 KO BMDCs. **(A).** Representative overlays of CD40, CD80 and CD86 expression on WT and TLR2 KO BMDCs stimulated with 5 µg/mL BcfA. Median fluorescence intensity (MFI) expression of **(B)**. CD40, **(C)**. CD80 and **(D)**. CD86 and percentage of cells expressing **(E)**. CD40, **(F)**. CD80 and **(G)**. CD86 on WT and TLR2 KO BMDCs stimulated with 1 µg/mL and 5 µg/mL BcfA for 20-24 hr. Heat-killed *B. pertussis* (HKBp) were used as positive controls. Mean ± SEM of triplicate wells is shown. ^*^, p<0.05, ^**^ by ANOVA. One experiment of 2.

Supplementary Figure 4 Gating strategy to identify lung CD11b^+^CD11c^+^ cells. Single cell suspensions from lungs were stained with antibodies specific for CD11b, CD11c, MHC Class II, CD40, CD80 and CD86. Live, single cells were gated as CD11b^+^CD11c^+^. This double positive population from WT and TLR4 KO lungs was evaluated for expression of MHC Class II, CD40, CD80 and CD86 as shown.
