# Supplementary figures and images for: The adjuvant BcfA activates antigen presenting cells through TLR4 and supports TFH and TH1 while attenuating TH2 gene programming
Source: Front Immunol. 2024 Aug 29;15:1439418. doi: 10.3389/fimmu.2024.1439418 (PMC11390363; doi:10.3389/fimmu.2024.1439418)

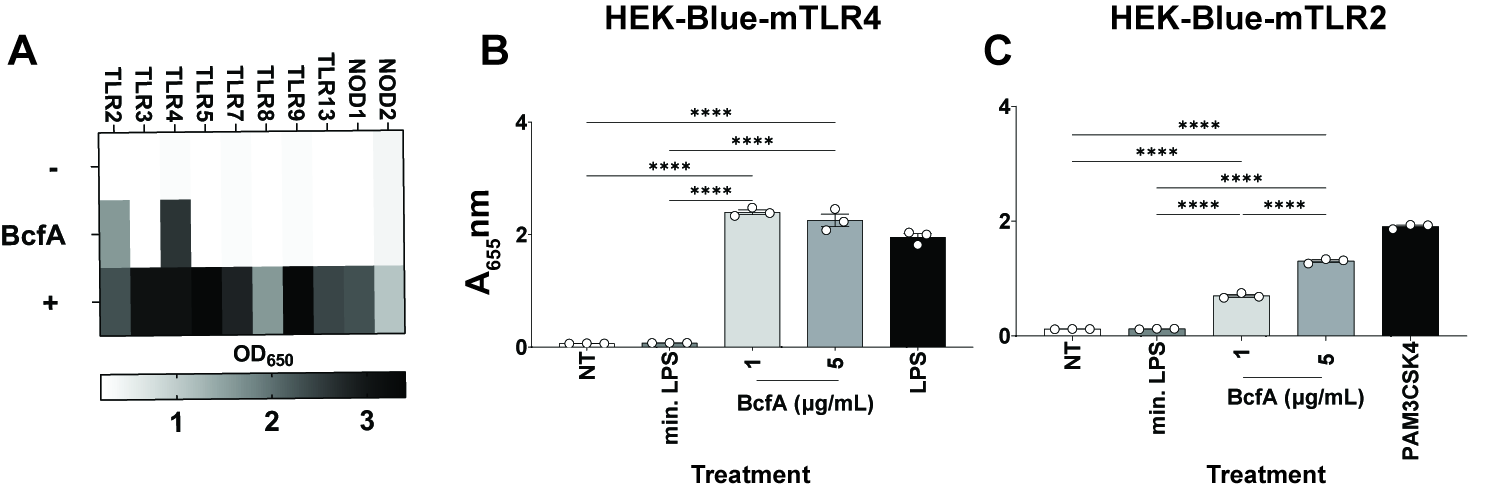

Supplement: Supplementary Figure 1 — BcfA activates BMDCs and TLR2 and TLR4 expressing reporter cell lines. (A). HEK-Blue™ cells expressing murine TLRs, and NOD1/2 were stimulated with BcfA (1µg/mL). SEAP reporter expression was detected using HEK-Blue™ Detection media. The average of duplicate wells is shown. (B). HEK-Blue-mTLR4 and (C). HEK-Blue-mTLR2 cells were stimulated with 1 and 5µg/mL of BcfA. NT = no treatment; min LPS = minimal LPS present in the BcfA preps. PAM3CSK4 and LPS were used as positive controls for TLR2 and TLR4 respectively. Mean ± SEM of triplicate wells is shown. ****, p<0.0001 by ANOVA. One experiment of 2. [file Image1.tif]

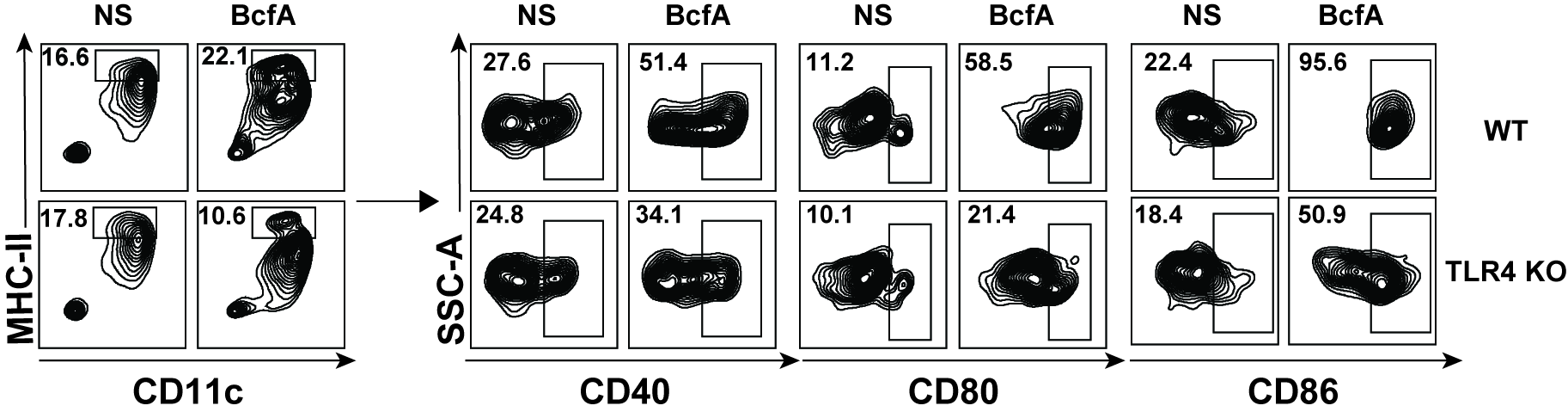

Supplement: Supplementary Figure 2 — Gating strategy to identify activated BMDCs. Differentiated BMDCs were stained with antibodies specific for CD11c, MHC Class II, CD40, CD80 and CD86. Live, single cells were gated as CD11c+MHC-IIhigh cells. This double positive population from WT and TLR4 KO cells was evaluated for expression of CD40, CD80 and CD86 as shown. [file Image2.tif]

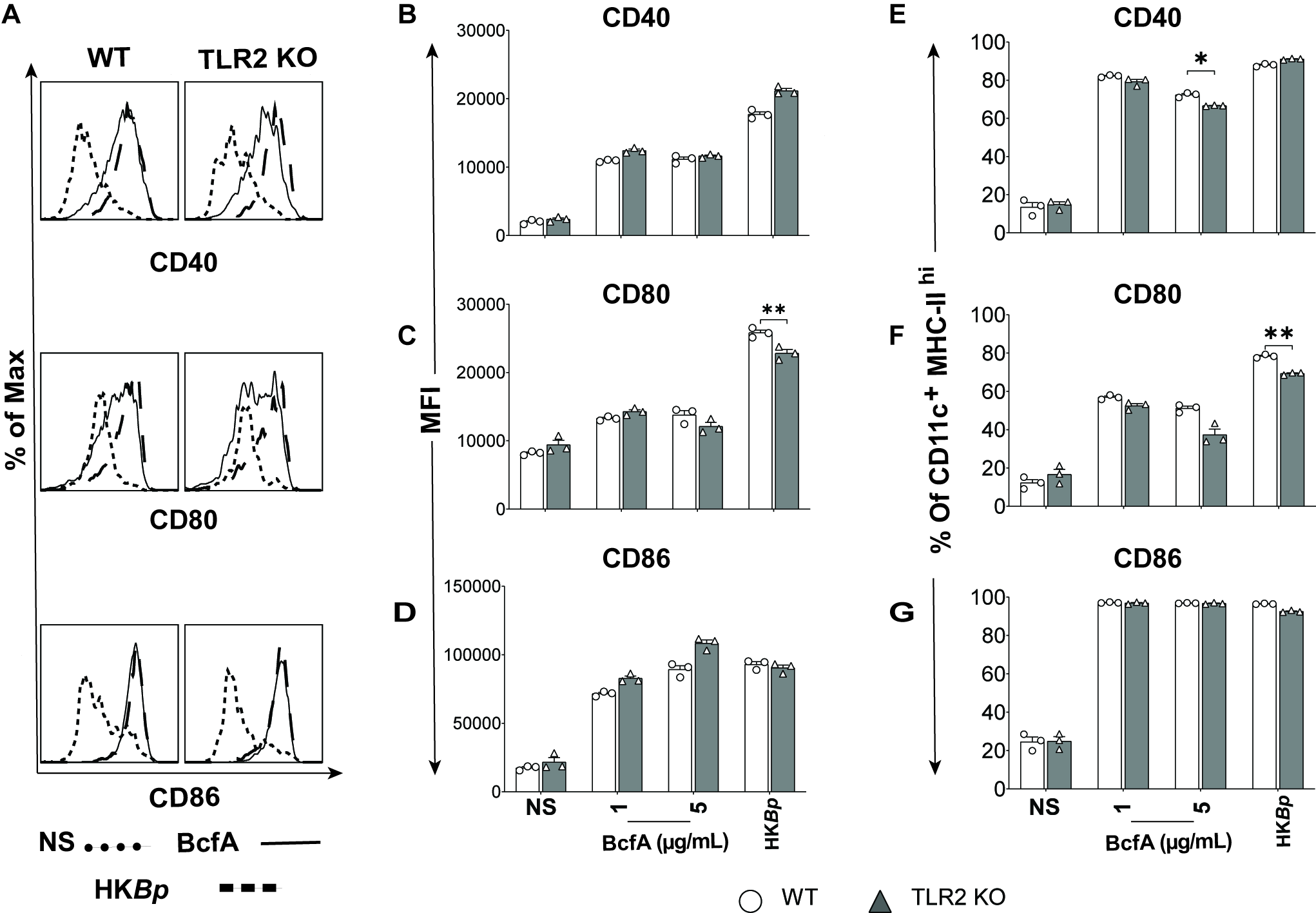

Supplement: Supplementary Figure 3 — Upregulation of costimulatory molecule expression is unchanged in TLR2 KO BMDCs. (A). Representative overlays of CD40, CD80 and CD86 expression on WT and TLR2 KO BMDCs stimulated with 5 µg/mL BcfA. Median fluorescence intensity (MFI) expression of (B). CD40, (C). CD80 and (D). CD86 and percentage of cells expressing (E). CD40, (F). CD80 and (G). CD86 on WT and TLR2 KO BMDCs stimulated with 1 µg/mL and 5 µg/mL BcfA for 20-24 hr. Heat-killed B. pertussis (HKBp) were used as positive controls. Mean ± SEM of triplicate wells is shown. *, p<0.05, ** by ANOVA. One experiment of 2. [file Image3.tif]

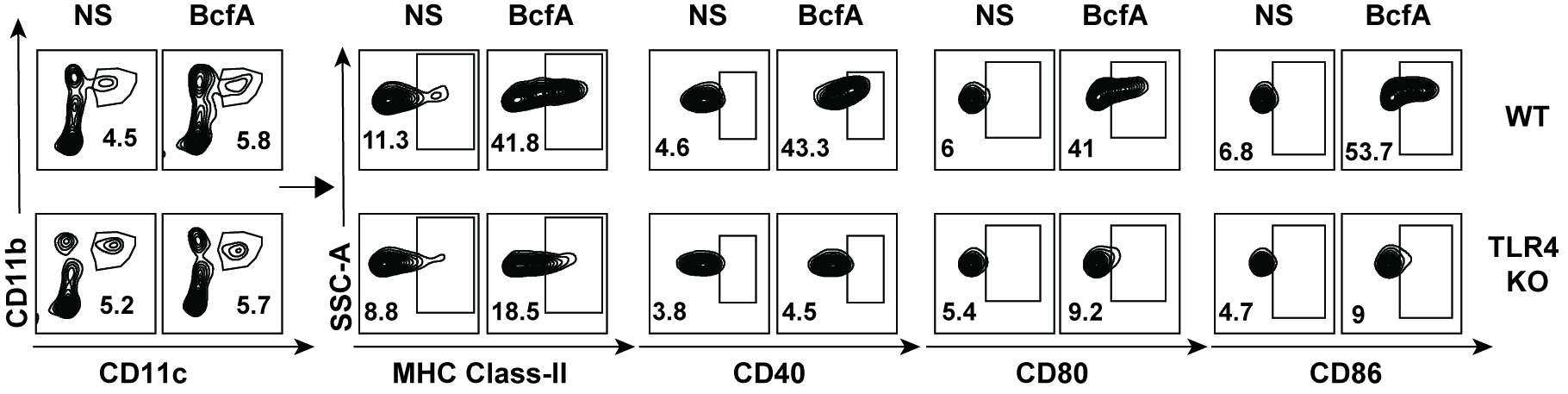

Supplement: Supplementary Figure 4 — Gating strategy to identify lung CD11b+CD11c+ cells. Single cell suspensions from lungs were stained with antibodies specific for CD11b, CD11c, MHC Class II, CD40, CD80 and CD86. Live, single cells were gated as CD11b+CD11c+. This double positive population from WT and TLR4 KO lungs was evaluated for expression of MHC Class II, CD40, CD80 and CD86 as shown. [file Image4.tif]
